# Effects of Daily Rhythm‐Aligned Training on Skeletal Muscle Adaptation in Older Adults: A Randomized Controlled Trial

**DOI:** 10.1002/jcsm.70390

**Published:** 2026-09-25

**Authors:** Fabienne Bruggisser, Soraya Ritter, Ralf Roth, Egbert T. Ritter, Denis Infanger, Romina Ledergerber, Timo Hinrichs, Frank A. J. L. Scheer, Christoph Handschin, Henner Hanssen, Raphael Knaier

**Affiliations:** ^1^ Department of Sport, Exercise and Health, Faculty of Medicine University of Basel Basel Switzerland; ^2^ Faculty of Sport and Health Sciences University of Jyväskylä Jyväskylä Finland; ^3^ Division of Sleep Medicine Harvard Medical School Boston Massachusetts USA; ^4^ Medical Chronobiology Program, Division of Sleep and Circadian Disorders, Departments of Medicine and Neurology Brigham, and Women's Hospital Boston Massachusetts USA; ^5^ Biozentrum University of Basel Basel Switzerland

**Keywords:** chronobiology, circadian rhythms, diurnal variation, exercise timing

## Abstract

**Background:**

Ageing is associated with declines in skeletal muscle mass and strength, increasing the risk of sarcopenia, functional impairment and reduced quality of life. Resistance training is an effective intervention to counteract these changes. However, the timing of training is rarely considered, even though physiological functions follow diurnal rhythms and individuals differ in the timing of peak physical performance. Therefore, this three‐arm, parallel‐group, randomized controlled superiority trial evaluated whether resistance training performed at an individual's peak time of day results in greater skeletal muscle adaptations than training performed at an individual's trough time of day or maintaining habitual lifestyle in older adults.

**Methods:**

In this single‐centre, double‐blind trial, participants completed standardized strength assessments at four time points (08:00, 12:00, 16:00 and 20:00) across 4 days to determine individual peak and trough performance times. Participants were then computer‐based block‐randomized (2:2:1) to (a) train at their individual peak time of day, (b) train at their trough time of day or (c) to a nontraining control group. The 12‐week intervention (comprising a 2‐week familiarization phase followed by 10 weeks of progressive training) consisted of two supervised whole‐body resistance training sessions per week, each consisting of five exercises, three sets and 8–15 repetitions, as well as one moderate‐intensity, 30‐min endurance cycling session at 60% of the individual's peak oxygen uptake.

The primary outcome was isometric midthigh pull strength; secondary outcomes included handgrip strength and appendicular lean mass index assessed by dual‐energy X‐ray absorptiometry. Between‐group differences were evaluated using baseline‐adjusted analyses of covariance including age, sex, body mass and training volume. The trial was prospectively registered (ClinicalTrials.gov: NCT06063135).

**Results:**

Of the 108 randomized participants (41% female; mean [standard deviation] age 67 years; body mass index 24.3 [2.7] kg·m^−2^), 93 were included in the analyses. Across all outcomes, effect estimates (peak vs. trough) were close to zero with confidence intervals overlapping zero (isometric midthigh pull strength: 0.06 N·kg^−1^ [95% confidence interval: −0.76 to 0.88]; handgrip strength: −0.21 kg·m^−2^ [−0.68 to 0.26]; appendicular lean mass index: 0.03 kg·m^−2^ [−0.09 to 0.15]), indicating no clear differences across groups. No intervention‐related serious adverse events were reported.

**Conclusion:**

Aligning resistance training with an individual's peak performance time did not provide evidence of superiority over trough conditions for skeletal muscle adaptations in older adults. These findings suggest that prescribing resistance training according to individualized diurnal performance timing is unlikely to provide clinically meaningful benefits over a range of training times.

## 1 Introduction

Life expectancy has increased continuously over recent decades. By 2050, more than 25% of the population in European and Northern American countries is expected to be 65 years or older [1]. Advanced age is a key risk factor for sarcopenia [2]. Despite the absence of a consensus definition, sarcopenia is commonly defined by age‐related declines in skeletal muscle mass, strength and function [2]. These changes emerge in the fifth decade of life and accelerate in subsequent decades [3]. Declines in skeletal muscle mass and strength play a central role in functional capacity, frailty and disability in older adults [4] and are key determinants of healthy ageing. Regular physical activity, particularly resistance training alone or combined with endurance training, preserves skeletal muscle mass and strength, attenuates sarcopenia progression [5] and confers broad health benefits [2, 4, 5]. Accordingly, guidelines for older adults recommend a multimodal exercise approach including strength, endurance, balance and flexibility training [6]. These guidelines follow the FITT principle (frequency, intensity, time and type) but do not consider the timing of exercise within the day.

Emerging evidence suggests that exercise timing may modulate physiological responses through interactions with the circadian system, which regulates endogenous ~24‐h rhythms in multiple biological functions, as well as with behavioural or environmental rhythms [7, 8, 9]. Both strength and endurance performance exhibit pronounced diurnal variation, with higher mean values generally observed in the afternoon and evening [10]. Accordingly, later‐day exercise has often been hypothesized to confer greater adaptations due to higher achievable exercise intensities [8]. However, longitudinal intervention studies provide only negligible evidence that exercising at any specific time of day yields superior performance or health‐related outcomes [11]. One potential explanation may be interindividual variability in the timing of peak performance. Although mean performance is generally higher later in the day, the time of day of peak strength [12] and endurance [13] performance varies substantially between individuals. Yet, all previous studies have neglected this interindividual variability and randomized participants to arbitrary times of day, most commonly 7:30 and 17:30 [11]. Aligning exercise with an individual's peak performance time may confer benefits beyond possibly enabling a higher acute training load: It may also optimize the biological processes underlying long‐term adaptation. Skeletal muscle contains its own molecular clock (CLOCK/BMAL1), which regulates key pathways involved in exercise adaptation, including substrate utilization, mitochondrial function, metabolic gene expression, mTOR‐mediated anabolic signalling and myokine secretion [14, 15]. Consequently, the same exercise session can elicit different molecular and metabolic responses depending on the time of day it is performed [16], while anabolic signalling after exercise also appears to be time‐of‐day dependent [17]. These findings raise the possibility that training during an individual's peak performance window may enhance long‐term strength and hypertrophic adaptations, rather than simply improving acute exercise performance.

The main objective of the present study was therefore to examine whether an individualized daily rhythm aligned exercise training approach, aligning exercise timing with the individual peak in the daily performance profile, would improve skeletal muscle adaptations more than with misaligned training. It was hypothesized that, in adults aged 60 to 80 years, 12 weeks of resistance training performed at the individual time of day of highest performance would result in greater improvements in skeletal muscle adaptations, encompassing both muscular strength and lean mass, compared with exercise performed at the time of day of lowest performance. Further objectives were to examine diurnal variation in strength performance and to assess whether exercise performed at different fixed times of day (morning, noon, afternoon and evening) resulted in different adaptations.

## 2 Material and Methods

### 2.1 Study Design and Randomization

The study was a single‐centre, double‐blind, three‐arm, parallel‐group, randomized controlled intervention trial conducted at the Department of Sport, Exercise and Health at the University of Basel, Switzerland, between November 2023 and June 2025. All assessments were performed in the laboratory under standardized conditions (humidity: 40%–55%; room temperature: 20°C–22°C). The study was funded by the Swiss National Science Foundation (grant number: PZ00P3_208999), approved by the Ethics Committee of Northwestern and Central Switzerland (EKNZ 2023‐01276) and prospectively registered on ClinicalTrials.gov (NCT06063135). The trial is reported in accordance with the Consolidated Standards of Reporting Trials guidelines [18]. Written informed consent was obtained from all participants prior to the initial assessment.

Using computer‐generated block randomization, participants were allocated in a 2:2:1 ratio to the peak group (i.e., training at the individual's peak time of day), the trough group (i.e., training at the individual's trough time of day) or the control group (i.e., maintaining their habitual lifestyle). Individual highest (peak) and lowest (trough) time of day were assessed in all participants as follows: Each participant performed strength tests at four different times of day (08:00, 12:00, 16:00 and 20:00) on four separate days with a minimum 24‐h rest period between tests. The order of testing times was identical for all participants; however, the time of day of the initial test was randomized.

### 2.2 Study Population

Eligible participants were males and females aged 60–80 years with a body mass index between 18.5 and 30 kg·m^−2^. Participants could be normoglycemic, prediabetic or diabetic, provided they were not treated with insulin or metformin. Eligibility was assessed using an online screening questionnaire, followed by a medical history interview and brief physical examination. Final eligibility was confirmed at the initial laboratory visit. Exclusion criteria primarily referred to medical conditions associated with a high risk of adverse events or inability to comply with the study procedures. Recruitment aimed for a balanced male‐to‐female ratio. Detailed eligibility criteria and procedures are provided in the Supporting Information (S2.1 and S2.2).

### 2.3 Exercise Intervention

Both exercise intervention groups underwent a structured, supervised exercise program three times per week for 12 weeks at the Department of Sport, Exercise and Health in Basel, Switzerland. Supervision was provided at a ratio of one exercise physiologist for every three to five participants. Exercise sessions were performed at the individually assigned peak or trough time of day. Training times were scheduled at 08:00, 12:00, 16:00 and 20:00. The exercise program comprised two weekly resistance training sessions and one weekly endurance training session.

Each resistance training session consisted of three sets of five exercises (single‐leg press [machine; performed on both sides], chest press [machine], deadlift [free weight; barbell], one‐arm cross body cable row [cable pulley; performed on both sides] and back squat [free weight; barbell]), with each session lasting between 45 and 65 min. Exercise order was fixed but the starting exercise rotated across sessions to minimize order effects. The first 2 weeks served as a familiarization period, during which participants performed three sets of 20 repetitions. From Weeks 3–12, participants were instructed to perform as many repetitions as possible with the prescribed load, until volitional failure, defined as the inability to complete another repetition with proper technique. The load remained constant across all three sets of each exercise. Therefore, the exercise‐specific volume was calculated by multiplying the load by the total number of repetitions performed in each session. Total session volume was subsequently derived by calculating the sum of all exercise‐specific volumes performed during a single session.

Load progression in training Weeks 3–12 followed a repetition‐based algorithm: If a participant exceeded 15 repetitions or failed to achieve at least eight in any of the three sets, the load was increased or decreased accordingly after the session. This repetition range and progression strategy was selected to elicit muscular hypertrophy and strength adaptations, consistent with established resistance training recommendations for older adults (i.e., moderate‐to‐high load, 8–15 repetitions to fatigue) [5, 19].

For each endurance training session, participants cycled continuously on an ergometer for 30 min at an intensity corresponding to 60% of their individual peak oxygen uptake, determined during baseline cardiopulmonary exercise testing. A chest strap was used to monitor heart rate during the training session, while an exercise physiologist controlled the intensity by adjusting the resistance of the ergometer.

Participants in the control group were instructed to maintain their habitual lifestyle throughout the 12‐week intervention period.

### 2.4 Outcome Measures

An overview of the assessed outcomes and monitored confounding factors is presented in Table S1. The primary outcome was maximal isometric strength assessed via the isometric midthigh pull (IMTP). This manuscript additionally reports the secondary outcomes of handgrip strength (HGS) and appendicular lean mass index (ALMI) assessed by dual‐energy X‐ray absorptiometry. Detailed descriptions of all secondary and exploratory outcomes in this trial, along with all monitored confounding factors, are provided in the Supporting Information (S2.4 and S2.5).

Standardization of pretesting behaviour was ensured by instructing participants to maintain normal dietary intake and adequate hydration during the 72‐h preceding testing. Strenuous physical activity and alcohol consumption were to be avoided for at least 24 h beforehand and caffeine intake for at least 4 h. Participants were further instructed to obtain sufficient sleep the night before testing. All instructions were communicated in advance, and compliance was confirmed prior to testing using predefined self‐report questions administered via the Research Electronic Data Capture platform.

### 2.5 Assessment of Participant Characteristics

Participants' height was measured to the nearest 0.5 cm using a stadiometer and body mass to the nearest 0.1 kg using a four‐segment bioelectrical impedance analyser (InBody 720; InBody Co. Ltd., Seoul, South Korea). Seated blood pressure and a resting 12‐lead electrocardiogram were obtained, followed by a physical examination. Participants then underwent maximal cardiopulmonary exercise testing with continuous electrocardiographic monitoring and breath‐by‐breath gas analysis as a means of preparticipation screening and exclusion of cardiopulmonary conditions. A detailed description of the cardiopulmonary exercise testing is provided in the Supporting Information (S2.4).

### 2.6 Outcome‐Specific Procedures

#### 2.6.1 Skeletal Muscle Strength

Strength was assessed via IMTP and HGS testing, each preceded by a familiarization session and standardized warm‐up. Relative maximal IMTP strength (N·kg^−1^) and relative HGS (kg·m^−2^) were derived as primary outcomes from the strength‐time curve. To capture overall daily strength, the maximum value at each of the four daily measurement times (08:00, 12:00, 16:00 and 20:00) was identified and averaged to derive a daily mean, for both pre‐intervention and post‐intervention assessments. Detailed testing procedures are provided in the Supporting Information (S2.6).

#### 2.6.2 Body Composition

Body composition was assessed via dual‐energy X‐ray absorptiometry (GE Lunar iDXA, GE Lunar Inc., Madison, WI). ALMI (kg·m^−2^) was calculated as appendicular lean soft tissue mass normalized to height squared, and body fat percentage was recorded as a descriptive characteristic. Further details are provided in the Supporting Information (S2.6).

### 2.7 Sample Size, Blinding and Harms

Sample size for the primary outcome was calculated a priori. As no preliminary IMTP data were available, effect size estimates were derived from published isometric knee extension studies in older adults [20, 21, 22], assuming an 8% greater strength gain in the peak versus trough group (*d* = 0.51). Using a two‐sided *α* of 0.05, 80% power and the method of Borm et al. [23] for pre‐adjusted analyses of covariance (pre–post correlation 0.65), required sample sizes were 36 per intervention group and 18 for the control group, increased to 42, 42 and 21, respectively, to account for 15% anticipated dropout.

Participants were allocated to peak, trough or control groups (2:2:1) using computer‐generated permuted block randomization with randomly varying block sizes. For the primary analysis (peak vs. trough), participants and supervising exercise physiologists were blinded to group allocation and trial hypotheses. Blinding was not feasible for the secondary (peak/trough vs. control) and tertiary (time‐of‐day) analyses, as training schedules were necessarily apparent. Strength and body composition assessments were performed by an investigator blinded to group allocation and uninvolved in training supervision; data were anonymized before statistical analysis to maintain statistician blinding.

Harms were monitored throughout the intervention, with adverse events recorded prospectively by supervising exercise physiologists at each session and assessment, and classified by type, severity and relationship to the intervention.

### 2.8 Statistical Analysis

Descriptive statistics were used to summarize participant characteristics, training adherence and training volume and load progression. Diurnal variation at pre‐intervention assessment was examined descriptively by comparing peak and trough values across testing times. Mean differences and corresponding 95% confidence intervals were calculated to characterize temporal variation.

Baseline‐adjusted analyses of covariance were conducted to examine between‐group differences in post‐intervention outcomes [24]. The primary model (Model 1), which was prespecified in the trial registration, included group allocation and the corresponding baseline value of the outcome. A second model (Model 2) additionally included age and sex for the outcomes IMTP strength and ALMI; age, sex and body mass for the outcome HGS. A third model (Model 3) further included training volume. For the analysis of time‐of‐day effects, randomization group (peak or trough) and training time of day (morning, noon, afternoon, or evening) were combined into a single categorical variable. In this parameterization, time‐of‐day was nested within the active randomization groups. Estimated marginal means were derived for each randomization–time‐of‐day combination. Further details on model specification and contrast construction are provided in the Supporting Information (S2.6).

Baseline values and continuous covariates were modelled using restricted cubic splines to allow for potential nonlinear associations. Between‐group differences were estimated using marginal means and pairwise contrasts and are reported as adjusted effect sizes with 95% confidence intervals. Effect sizes were calculated in the original units and standardized to the baseline standard deviation of the respective outcome. Model assumptions were verified graphically using normal Q–Q plots and Tukey–Anscombe plots.

Additionally, exploratory analyses were conducted to examine potential congruency effects between training and testing time, defined as assessments performed at the same time of day as training (congruent) or at a different time of day (incongruent).

All analyses followed a modified intention‐to‐treat principle, with participants analysed according to their original randomization regardless of level of adherence to the intervention. Missing data were assumed to be missing at random; given the low drop‐out rates, no imputation was performed and analyses were based on complete cases, excluding participants only from models where the relevant pre‐intervention or postintervention measurement was missing. The number of participants contributing to each analysis is reported in the corresponding results tables.

Statistical analyses were performed in R (version 4.5.2; R Foundation for Statistical Computing, Vienna, Austria). Pairwise contrasts within each model were adjusted for multiple comparisons using a multivariate t (mvt) distribution‐based adjustment. Details on analysed sample, randomization, restricted cubic splines and further R packages used are provided in the Supporting Information (S2.6).

## 3 Results

### 3.1 Study Sample

**FIGURE 1:**
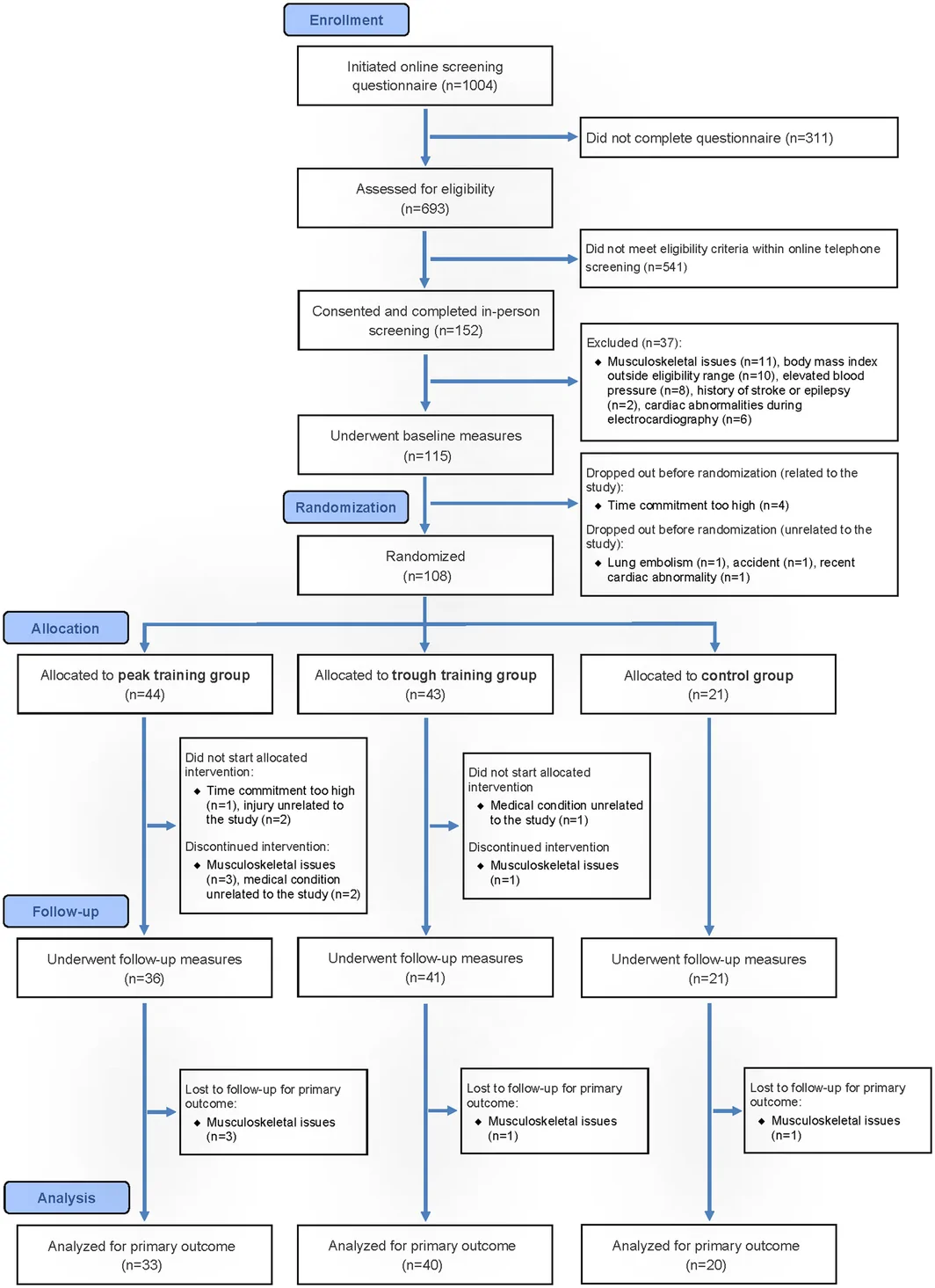
Participant flow diagram.

Of the 115 participants who underwent baseline assessments, 108 were randomized, of whom 93 were included in the outcome analyses (Figure 1). Baseline characteristics of the randomized participants are presented in Tables 1 and S2, which are stratified by peak–trough–control randomization groups and time‐of‐day‐specific groups, respectively. A higher proportion of males was enrolled in the study, resulting in unequal sex distributions across randomization groups. The intervention‐related dropout rates between randomization and post‐intervention assessments were 14% in the peak group, 5% in the trough group and 5% in the control group. Of the 15 participants who withdrew, reasons included musculoskeletal complaints (*n* = 9), medical conditions or injuries unrelated to the study (*n* = 5) and excessive time commitment (*n* = 1), with no systematic differences between groups. Dropouts in the peak group were predominantly female (8 females, 3 males), while dropout rates were more balanced in the trough (1 female, 2 males) and control (0 females, 1 male) groups. The dropout rates for all reasons of withdrawal across time‐of‐day‐specific groups were 0%, 11%, 29% and 23% in the morning, noon, afternoon and evening groups, respectively. Detailed reasons for withdrawal by time‐of‐day‐specific groups are provided in the Supporting Information (S3.2). No intervention‐related serious adverse events were reported.

**TABLE 1:** Baseline characteristics of the randomized participants.

Characteristics	Total	Peak	Trough	Control
Sex, male/female (*n*)	64/44	22/22	28/15	14/7
Age (years)	67 (5) [60–80]	67 (5) [60–78]	67 (5) [60–78]	66 (7) [60–80]
Body mass (kg)	72.5 (12.4) [50.2–99.4]	71.5 (13.2) [50.2–97.1]	74.3 (12.6) [52.6–99.4]	70.6 (10.0) [53.0–88.4]
Body height (cm)	172 (8) [153–190]	171 (9) [156–190]	173 (7) [155–185]	172 (8) [153–184]
BMI (kg·m^−2^)	24.3 (2.7) [19.0–29.8]	24.2 (2.7) [19.0–28.7]	24.7 (2.9) [19.4–29.8]	23.7 (2.1) [20.7–28.7]
Body fat content (%)	30.8 (6.9) [10.3–46.7]	31.6 (7.3) [16.7–46.7]	31.2 (6.4) [16.5–43.7]	28.3 (6.8) [10.3–38.1]

*Note:* Data are mean (standard deviation) [range] unless noted otherwise.

Abbreviations: BMI, body mass index; Control, control group maintaining their current lifestyle; Peak, intervention group training at their individual peak time of day; Trough, intervention group training at their individual trough time of day.

### 3.2 Training Adherence and Progression

Adherence to resistance training was high and comparable between groups (peak: 91% [mean 21.8 of 24 sessions; range 16–24], trough: 93% [22.4 of 24 sessions; 16–24]). Endurance training adherence was similarly high (peak: 94% [11.3 of 12 sessions; 8–12], trough: 94% [11.2 of 12 sessions; 8–12]). When stratified by time‐of‐day‐specific groups, adherence remained high for both resistance (morning: 95% [22.7 of 24 sessions; 16–24], noon: 91% [21.8 of 24 sessions; 18–24], afternoon: 91% [21.8 of 24 sessions; 16–24], evening: 92% [22.1 of 24 sessions; 17–24]) and endurance training (morning: 92% [11.0 of 12 sessions; 8–12], noon: 92% [11.1 of 12 sessions; 8–12], afternoon: 99% [11.9 of 12 sessions], evening: 94% [11.3 of 12 sessions; 9–12]), with no systematic differences between groups.

**FIGURE 2:**
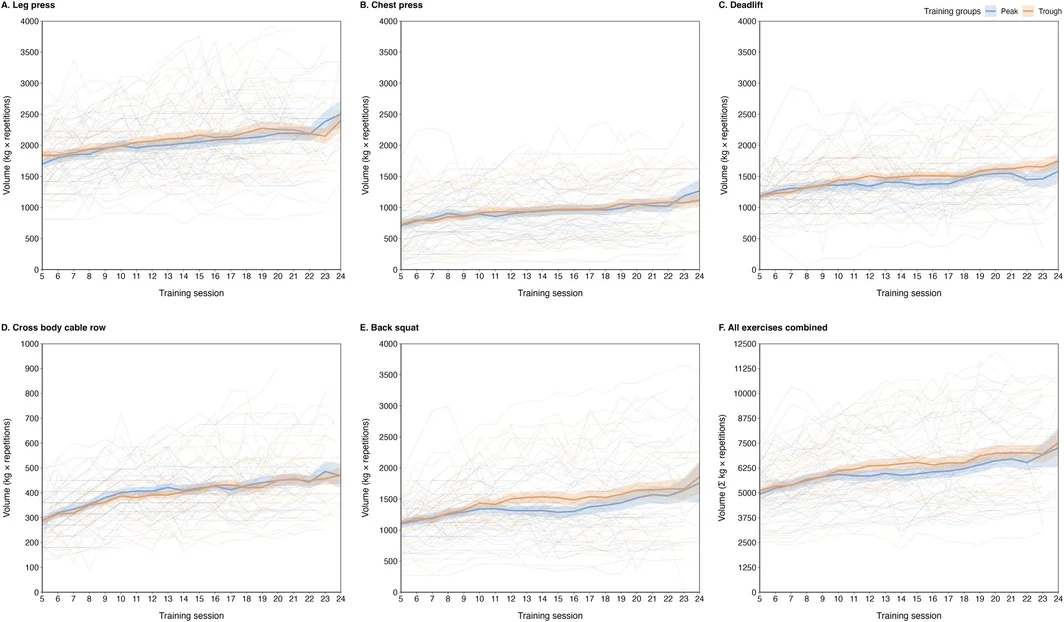
Exercise‐specific volume (panels A–E) and total session volume (panel F) across resistance training sessions for the peak (training at their individual peak time of day) and the trough (training at their individual trough time of day) groups. Exercise‐specific volume was calculated as load × repetitions; for bilateral exercises (leg press and cross body cable row), volume was calculated for a single side only. Total session volume was computed as the sum of all exercise‐specific volumes per session. Solid lines indicate group means, shaded areas reflect variability and thin lines represent individual participant trajectories.

Exercise‐specific volume (Figure 2A–E) and total session volume (Figure 2F) increased progressively throughout the intervention in both peak and trough groups, with comparable patterns observed across time‐of‐day‐specific groups (Figure S1). Absolute external load per exercise increased throughout the intervention in both peak and trough groups (Figure S2) and across time‐of‐day‐specific groups (Figure S3). Despite substantial interindividual variability, the magnitude and direction of mean changes in volume and absolute load were similar between groups.

### 3.3 Diurnal Variation

At pre‐intervention assessment, peak relative IMTP strength most frequently occurred in the afternoon (32%) and evening (33%), whereas trough values were predominantly observed at noon (40%) and in the morning (29%). Mean relative IMTP strength differed between peak and trough times of day (mean: 25.9 (standard deviation: 4.0) and 23.3 (3.6) N·kg^−1^, respectively), with a mean difference of 2.6 (1.4) N·kg^−1^ [95% confidence interval: 2.3–2.9]. A comparable pattern was observed for HGS, with a mean difference of 1.4 (0.8) kg·m^−2^ [1.3–1.6]. The distribution across all four test times and stratified groups (all participants, peak–trough–control randomization, and time‐of‐day‐specific groups) is illustrated in Figure S4, confirming a modest but consistent temporal pattern with generally higher performance later in the day. However, substantial interindividual variability is evident, as reflected by the wide and overlapping individual trajectories across time points.

### 3.4 Training‐Induced Adaptations

**FIGURE 3:**
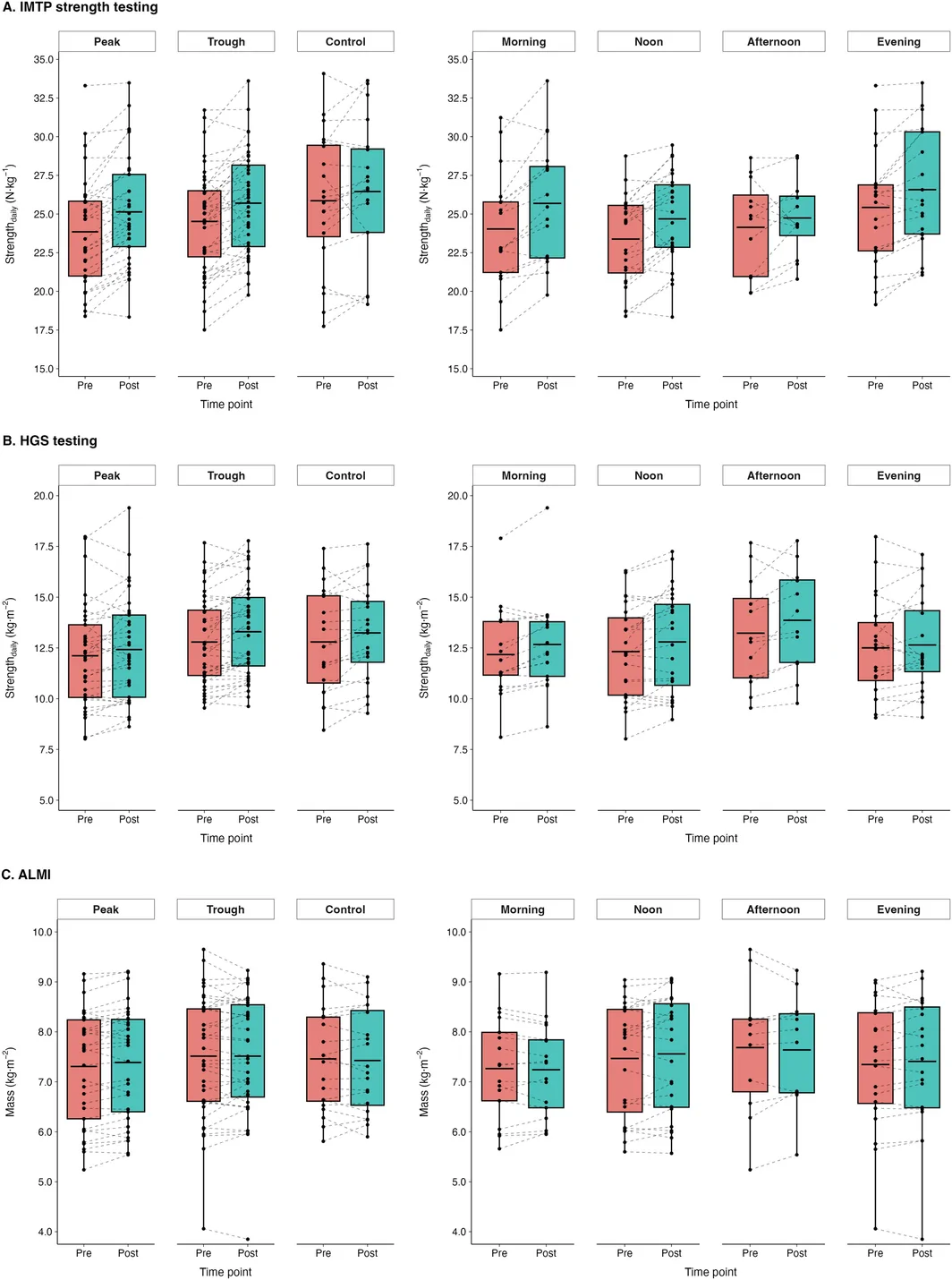
Relative mean daily isometric midthigh pull (IMTP) strength (A), relative mean daily handgrip strength (HGS) (B) and appendicular lean mass index (ALMI) (C) shown pre‐intervention and post‐intervention, stratified by peak–trough–control randomization and time‐of‐day‐specific allocation. ‘Peak’ and ‘Trough’ denote intervention groups training at their individual peak or trough time of day, respectively, and ‘Control’ represents the control group maintaining their current lifestyle. ‘Morning’, ‘Noon’, ‘Afternoon’ and ‘Evening’ represent intervention groups training at 08:00, 12:00, 16:00 and 20:00, respectively. Time points are ‘Pre’ (before the training intervention or control phase) and ‘Post’ (after the training intervention or control phase). ‘Strength_daily_’ represents the mean of each participant's maximal strength values obtained at 4 measurement times (08:00, 12:00, 16:00 and 20:00). Individual participant changes are shown with dashed lines, with boxplots representing the distribution of values at each time point.

Descriptive pre‐intervention and post‐intervention values and changes over time for mean daily relative IMTP strength and HGS, as well as for ALMI, are shown in Figure 3 (stratified by peak–trough–control randomization groups and time‐of‐day‐specific groups) and Figure S5 (peak–trough groups, stratified by sex). Relative mean daily IMTP strength and HGS increased in all groups from pre‐intervention to postintervention (Figure 3). Pre–post improvements were evident in both females and males across peak and trough groups, with similar patterns of change despite sex differences in absolute strength levels (Figure S5).

**FIGURE 4:**
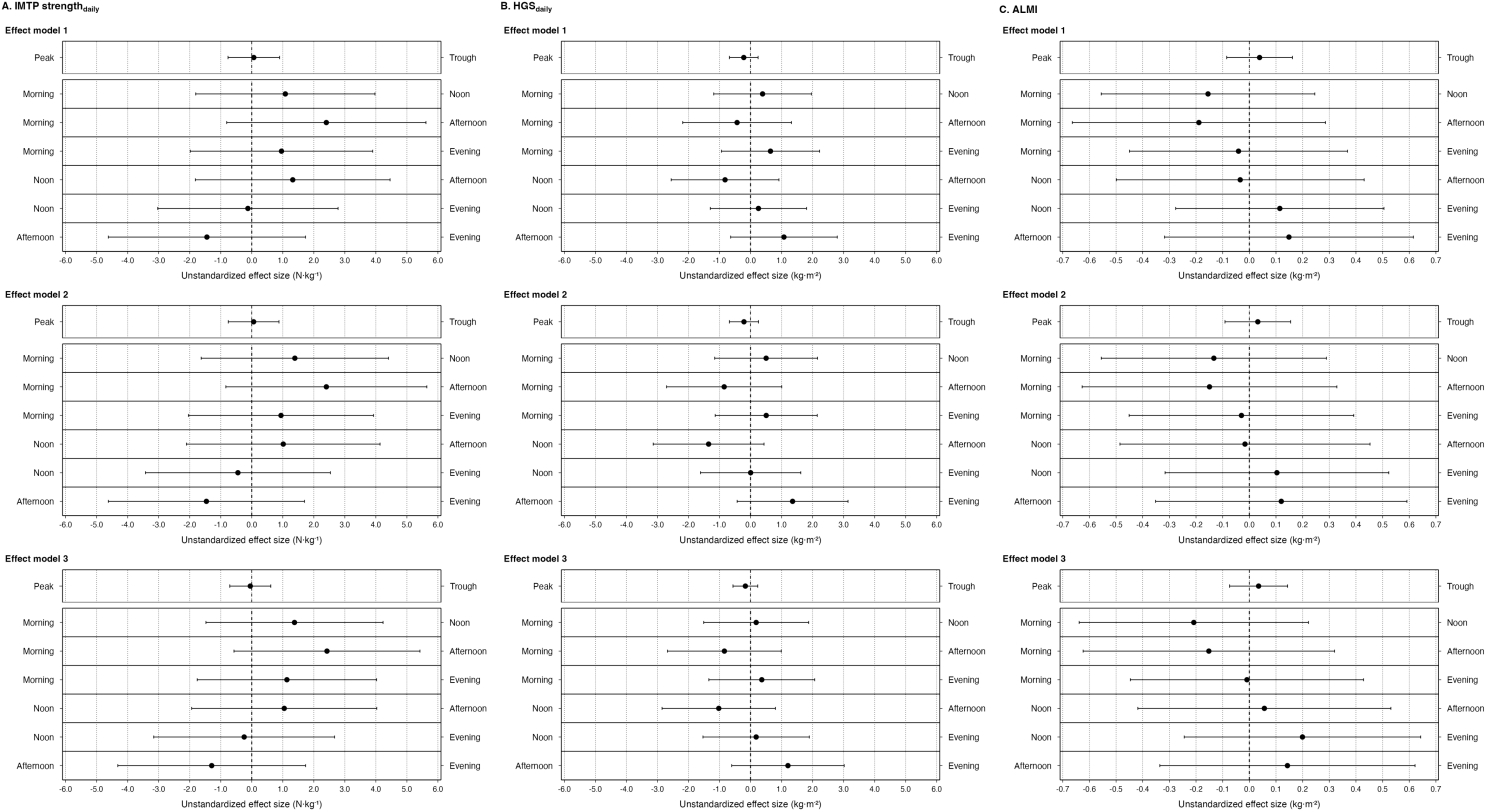
Between‐group effect sizes with 95% confidence intervals are shown for relative daily mean isometric midthigh pull strength (IMTP strength_daily_) (A), relative daily mean handgrip strength (HGS_daily_) (B) and appendicular lean mass index (ALMI) (C). Effect sizes are expressed in the original outcome units (N·kg^−1^ for IMTP strength, kg·m^−2^ for HGS and ALMI). Model 1 adjusts for baseline values and group allocation; Model 2 additionally includes age and sex for the outcomes IMTP strength and ALMI and age, sex and body mass for the outcome HGS; Model 3 further includes training volume. ‘Peak’ and ‘Trough’ denote training groups randomized to train at individual peak or trough time of day, respectively. ‘Morning’, ‘Noon’, ‘Afternoon’ and ‘Evening’ represent intervention groups training at 08:00, 12:00, 16:00 and 20:00, respectively. Points represent unstandardized effect sizes; horizontal bars indicate 95% confidence intervals.

In adjusted post‐intervention comparisons, differences between the peak and trough groups were small, with effect sizes close to zero across all models for relative mean daily IMTP strength (≈ 0.07 N·kg^−1^), relative mean daily HGS (≈ − 0.20 kg·m^−2^), and ALMI (≈ 0.04 kg·m^−2^) (Figure 4 and Table S3). Confidence intervals were largely overlapped zero for IMTP strength, HGS, and ALMI, indicating no clear differences in adaptations between peak and trough training groups. Across time‐of‐day‐specific group contrasts, relative mean daily IMTP strength showed the largest deviations for the morning‐afternoon comparison (up to ≈ 2.40 N·kg^−1^), whereas the remaining contrasts were inconsistent and close to zero (Figure 4, Table S3). For HGS, time‐of‐day‐specific contrasts showed no consistent pattern, with the largest deviation observed for the afternoon‐evening comparison (up to ≈ 1.35 kg·m^−2^ in Models 2 and 3). However, confidence intervals were wide and overlapped zero across contrasts, indicating substantial uncertainty. For ALMI, pairwise contrasts were consistently close to zero (≤ 0.04 kg·m^−2^), with wide confidence intervals across statistical models, indicating no relevant differences between groups. Standardized effect sizes (Table S4) showed the same pattern, with all values close to zero and confidence intervals overlapping zero, indicating trivial to small and uncertain effects across all contrasts. Comparisons between training groups and the control group yielded similarly small effect sizes (Tables S3 and S4).

In contrast to the primary analyses based on daily mean values, further exploratory analyses descriptively examined time‐of‐day‐specific pre–post changes in IMTP strength and HGS (Figure S6). No consistent evidence of a benefit of congruent versus incongruent training and testing conditions for performance‐related outcomes was observed. Pre–post changes were broadly comparable across test times of day within each time‐of‐day‐specific allocation group, with substantial interindividual variability.

## 4 Discussion

Contrary to our hypothesis, training at the individualized time of day of peak performance did not result in superior adaptations compared to training at the trough time of day. Furthermore, training performed at fixed times of day (morning, noon, afternoon, evening) did not result in different training adaptations. These findings do not provide evidence that optimizing the timing of resistance training enhances skeletal muscle adaptations in adults aged 60–80 years.

### 4.1 The Role of Circadian Rhythms in Exercise Physiology

Our pre‐intervention assessment confirmed that this diurnal pattern is also present in older adults. However, despite these diurnal fluctuations in baseline performance, aligning training with individual peak performance time did not result in superior training adaptations. These findings align with previous longitudinal studies reporting similar strength and hypertrophic adaptations irrespective of whether resistance training is performed in the morning or later in the day [8, 11, 25, 26, 27]. Nevertheless, the literature remains inconsistent, as some studies have reported greater adaptations when training is performed later in the day or when training and testing occur at the same time of day [11, 28]. Importantly, most existing studies have been conducted in young, healthy adults and frequently include predominantly male participants, limiting generalizability to older populations or females [11, 25, 26, 28]. Evidence in older adults remains scarce. The few available studies have reported significant improvements in strength following resistance training, but without consistent differences between morning and afternoon or evening training [27, 29].

There are several possible explanations for the inconsistent findings across studies, which may relate to the methodological approach used in most time‐of‐day training interventions. First, all previous trials [11] only investigated two times of day resulting in a high chance of missing the time of day that has the most beneficial health effects. In contrast, in this trial, we investigated four different times of day. Second, in contrast to most previous trials, this trial included the most rigorous and comprehensive monitoring of confounding factors and gold‐standard methods to assess the outcomes. Third, and most importantly, participants are typically randomized to fixed training times, which fail to account for substantial interindividual variability in the timing and magnitude of daily performance peaks [12, 13]. In the present cohort, mean diurnal variation in strength was approximately 11%, with individual amplitudes ranging from 1% to 34%. Consequently, participants assigned to the same training time of day may train at markedly different relative performance states.

To address this, the present trial implemented an individualized circadian exercise approach in which participants trained either close to the individual peak or the trough of daily strength performance. To our knowledge, this is the first randomized controlled trial to test such a peak–trough design in resistance training. However, despite this individualized approach, no superiority of peak training was observed. This discrepancy may reflect the highly decentralized nature of human chronobiology. While the suprachiasmatic nucleus acts as the central pacemaker, numerous peripheral tissues, including skeletal muscle, possess semiautonomous molecular clocks that regulate local physiological processes [9, 30, 31]. Importantly, these systems do not necessarily operate in synchrony. The physiological systems driving acute performance (e.g., neuromuscular activation or thermoregulatory rhythms) may peak at different times of day than those regulating cellular processes such as muscle protein synthesis and tissue repair [9]. Such temporal dissociation may explain why aligning training with maximal strength output did not translate into greater long‐term adaptations. Moreover, although absolute strength varies across the day, resistance training performed to volitional failure likely results in a comparable relative training stimulus across time points, which may further explain the absence of time‐of‐day‐specific differences in long‐term adaptations. Training adaptations are likely influenced by multiple interacting physiological processes rather than by a single circadian marker. Consequently, reducing circadian exercise strategies to simplified concepts such as “training at peak performance time” or interpreting timing effects solely through individual physiological indicators (e.g., core body temperature) may not adequately capture the underlying biological mechanisms.

### 4.2 Methodological Considerations

Several methodological considerations should be taken into account when interpreting these findings. First, individualized peak and trough times of day were identified for each participant. However, the magnitude of diurnal variation differed between individuals, resulting in a larger physiological contrast between peak and trough conditions in some participants than in others. Such interindividual variability in the amplitude of diurnal performance rhythms is a well‐recognized physiological phenomenon and is consistent with previous observations in both strength and endurance performance [12, 13]. Consequently, participants with smaller diurnal amplitudes would be expected to experience a smaller physiological contrast between training conditions, which may have attenuated between‐group differences. However, this fact was considered in the sample size calculation. Because each participant's peak and trough times were derived from a single four‐day profiling period, day‐to‐day variability in strength performance means that these times represent an estimate rather than a definitive determination, which may have weakened the intended physiological contrast. Second, repeated exposure to exercise at a consistent time of day may have resulted in temporal acclimatization. Physiological and neural systems may adapt to regular training stimuli at a given time of day, potentially diminishing initial circadian differences in performance [32]. Third, characteristics of the study population and intervention may have reduced the ability to detect subtle timing‐specific effects. Participants were relatively healthy and high‐functioning, with HGS values above the population average for their age and sex [3]. Moreover, early resistance‐training adaptations in older adults are predominantly neural rather than hypertrophic [5], while hypertrophic adaptations develop gradually and are typically small. Together with the initial 2‐week familiarization period, which left approximately 10 weeks of progressive overload, this may have reduced sensitivity to detect small differences in muscle hypertrophy. Consequently, minor timing‐specific differences in ALMI may have remained within the measurement variability of dual‐energy X‐ray absorptiometry [33, 34]. Fourth, characteristics of the strength assessments may also have influenced the results. Owing to its isometric nature, the IMTP may not fully capture all task‐specific adaptations elicited by dynamic isotonic resistance training [35]. Furthermore, maximal IMTP performance requires sufficient HGS to stabilize the bar. Because HGS is unlikely to improve substantially in response to predominantly lower body training, limitations in distal force transmission could partially mask genuine improvements in lower limb strength [36].

Although habitual sleep timing and chronotype were not used to prescribe training times, ageing is associated with reduced circadian rhythm amplitude [37]. Additionally, chronotype variability and sex differences in chronotype diminish after approximately 60 years of age [38]. Consequently, potential confounding factors related to circadian preference were likely minimized, though some residual interindividual variability in circadian physiology cannot be ruled out. While biological sex and menopausal status influence circadian physiology [39], these factors are less likely to materially affect the primary comparison in this older cohort. However, the unequal sex distribution between groups and the absence of information on menopausal status may have introduced residual variability. As a single‐centre trial of healthy, independently living older adults, the findings may not generalize to frail, sarcopenic, multimorbid, or institutionalized populations. The study was prospectively powered to detect the pre‐specified between‐group effect size for the primary outcome (*d* = 0.51). Although larger sample sizes might detect smaller between‐group differences, the observed point estimates were consistently close to zero, suggesting that any undetected effects are unlikely to be clinically meaningful or sufficient to justify recommendations to alter habitual training time.

Despite these methodological considerations, the present trial has several strengths, including its randomized controlled design, individualized time‐of‐day allocation based on repeated performance testing and the use of the IMTP as a safe, highly standardized and reproducible measure of maximal force production well suited to repeated testing in older adults. Together with the comprehensive assessment of strength and body composition outcomes, these design features enabled a robust evaluation of training time effects.

## 5 Conclusion

In this trial, aligning resistance training with an individual's peak performance time of day did not enhance skeletal muscle adaptations compared with training at the trough of daily performance. These findings suggest that prescribing resistance training according to an individual's peak performance time is unlikely to provide clinically meaningful additional benefits over training at the trough of daily performance in healthy older adults. Additional comparisons between fixed training times (morning, noon, afternoon and evening) likewise showed no consistent evidence that a specific time of day confers superior skeletal muscle adaptations. These findings suggest that resistance training can be effectively performed across different times of day in older adults, supporting flexible scheduling of exercise sessions to promote adherence without compromising muscular adaptations.

## Funding

F.B., S.R., R.L. and R.K. (principal investigator) were funded by the Swiss National Science Foundation, grant no. PZ00P3_208999.

## Ethics Statement

The present work follows the ethical standards for authorship and publishing in the *Journal of Cachexia, Sarcopenia and Muscle* [40].

## Conflicts of Interest

F.A.J.L. Scheer has served on the Board of Directors for the Sleep Research Society and has received consulting fees from the University of Alabama at Birmingham, Morehouse School of Medicine, and Salk Institute for Biological Studies. F.A.J.L. Scheer's interests were reviewed and managed by Brigham and Women's Hospital and Partners HealthCare in accordance with their conflict of interest policies. F.A.J.L. Scheer's consultancies are not related to the current work. The other authors declare no conflicts of interest.

## Supporting information




**Table S1:** Overview of outcome variables, assessment methods and assessment time points.
**Table S2:** Baseline characteristics of the time‐of‐day‐specific groups.
**Figure S1:** Exercise‐specific volume (A–E) and total session volume (F) across resistance training sessions for the morning (training at 08:00), noon (training at 12:00), afternoon (training at 16:00) and evening (training at 20:00) groups. Exercise‐specific volume was calculated as load × repetitions; for bilateral exercises (leg press and cross body cable row), volume was calculated for a single side only. Total session volume was computed as the sum of all exercise‐specific volumes per session. Solid lines depict group means, shaded areas indicate variability and thin lines represent individual participant trajectories.
**Figure S2:** Absolute training loads for leg press (A), chest press (B), deadlift (C), cross body cable row (D) and back squat (E) across time points for the peak (training at their individual peak time of day) and trough (training at their individual trough time of day) groups. Time points include T1 (first resistance training session following the 2‐week familiarization period), T2 (midpoint of the resistance training phase, corresponding to 50% of each participant's individual maximum number of completed resistance training sessions) and T3 (final resistance training session). Individual participant trajectories are shown with dashed lines, with boxplots representing the distribution of training loads at each time point.
**Figure S3:** Absolute training loads for leg press (A), chest press (B), deadlift (C), cross body cable row (D), and back squat (E) are shown across time points for the morning (training at 08:00), noon (training at 12:00), afternoon (training at 16:00) and evening (training at 20:00) groups. Time points include T1 (first resistance training session following the 2‐week familiarization period), T2 (midpoint of the resistance training phase, corresponding to 50% of each participant’s individual maximum number of completed resistance training sessions) and T3 (final resistance training session). Individual participant trajectories are shown with dashed lines, with boxplots representing the distribution of training loads at each time point.
**Figure S4:** Diurnal variation in relative isometric midthigh pull (IMTP) strength (A) and relative handgrip strength (HGS) (B) across test times of day. Data are shown for all participants, for peak–trough–control randomization groups, and for time‐of‐day‐specific groups. Boxplots depict the distribution of values at each test times of day, with individual participant trajectories shown as dashed lines. Test times of day are morning (M), noon, (N), afternoon (A) and evening (E).
**Figure S5:** Relative mean daily isometric midthigh pull (IMTP) strength (A), relative mean daily handgrip strength (HGS) (B) and appendicular lean mass index (ALMI) (C) shown pre‐intervention and postintervention, stratified by peak–trough randomization and sex. ‘Peak’ and ‘Trough’ denote intervention groups training at their individual peak or trough time of day, respectively. Time points are ‘Pre’ (before the training intervention or control phase) and ‘Post’ (after the training intervention or control phase). ‘Strength_daily_’ represents the mean of each participant's maximal strength values obtained at four measurement times (08:00, 12:00, 16:00 and 20:00). Dashed lines depict individual participant changes between time points; boxplots summarize the distribution at each time point.
**Table S3:** Baseline‐adjusted between‐group differences reported as effect sizes derived from analyses of covariance.
**Table S4:** Baseline‐adjusted between‐group differences reported as standardized effect sizes derived from analyses of covariance.
**Figure S6:** Pre–post changes in relative isometric midthigh pull (IMTP) strength (A) and relative handgrip strength (HGS) (B) stratified by time‐of‐day‐specific allocation and test times of day. The figure illustrates whether participants who trained at a given time of day (morning, noon, afternoon or evening) showed larger improvements when tested at the corresponding time of day. The control group (Control), which maintained their habitual lifestyle, was also included in the analysis. Boxplots representing the distribution of values at each time point. Test times of day are morning (M), noon, (N), afternoon (A) and evening (E).

## Data Availability

The datasets generated and analysed during the current study are available from the corresponding author on request. Once analyses of all other secondary outcomes from this study are completed, data will be provided on a data repository.
