# Supplementary material for: Effects of Daily Rhythm‐Aligned Training on Skeletal Muscle Adaptation in Older Adults: A Randomized Controlled Trial
Source: J Cachexia Sarcopenia Muscle. 2026 Sep 25;17(5):e70390. doi: 10.1002/jcsm.70390 (PMC13614912; doi:10.1002/jcsm.70390)
Supplement: Supplementary file 1 [file JCSM-17-e70390-s001.docx]

*Supporting Information – Journal of Cachexia, Sarcopenia and Muscle*

**Effects of Daily Rhythm-Aligned Training on Skeletal Muscle Adaptation in Older Adults: A Randomized Controlled Trial**

Fabienne Bruggisser^a^, Soraya Ritter^a^, Ralf Roth^a^, Egbert T. Ritter^a^, Denis Infanger^a^, Romina Ledergerber^a^, Timo Hinrichs^b^, Frank A.J.L. Scheer^c,d^, Christoph Handschin^e^, Henner Hanssen^a^, Raphael Knaier^a,*^

^a^Department of Sport, Exercise and Health, Faculty of Medicine, University of Basel, Basel, Switzerland.

^b^Faculty of Sport and Health Sciences, University of Jyväskylä, Jyväskylä, Finland.

^c^Division of Sleep Medicine, Harvard Medical School, Boston, MA, USA.

^d^Medical Chronobiology Program, Division of Sleep and Circadian Disorders, Departments of Medicine and Neurology, Brigham, and Women’s Hospital, Boston, MA, USA.

^e^Biozentrum, University of Basel, Basel, Switzerland.

^*^Corresponding author.

*E-mail address*: [raphael.knaier@unibas.ch](mailto:raphael.knaier@unibas.ch) (R. Knaier)

**S2 Material and Methods**

**S2.1 Eligibility Criteria**

Exclusion criteria included current smoking or smoking cessation within the past ten years; participation in structured resistance training during the previous six months; ongoing night-shift work; irregular sleep-wake patterns (> two hours variation); and travel across more than two time zones within the preceding four weeks. Individuals receiving anti-diabetic medication were included with exception of metformin, as prior evidence suggests that it may reduce the muscle building effects of resistance training and alter muscle transcriptomic responses in older adults [1,2]. Additional exclusion criteria included pregnancy or lactation; participation in another clinical trial within the previous four weeks; and inability to comply with study procedures (e.g., due to language barriers, cognitive impairment or psychological disorders). Participants were also excluded if they had contraindications for resistance or endurance testing and training, including uncontrolled hypertension (systolic >170 mmHg or diastolic >100 mmHg), unstable cardiovascular disease, severe arrhythmias, valvular disease, decompensated cardiovascular disease, ongoing cancer treatment, acute infection, or musculoskeletal/orthopedic limitations.

The rationale for the selected age range is as follows: (i) the prevalence of sarcopenia is relatively low below the age of 60 years; and (ii) individuals above the age of 80 years exhibit a higher burden of comorbidities. Further methodological advantages of this age range include: (i) a high proportion of retired individuals, allowing for greater scheduling flexibility; (ii) a predominance of postmenopausal females; (iii) a low prevalence of extreme late chronotypes; and (iv) minimal sex-related differences in chronotype at this age. Collectively, these factors increased recruitment and reduced potential confounding factors, including the menstrual cycle and sleep restriction in late chronotypes undergoing morning exercise testing.

The rationale for the body mass index range is as follows: (i) to obtain generalizable results that include both normal and overweight participants; (ii) participants within the normal body mass index range can already present an increased risk of cardiometabolic diseases; and (iii) sarcopenia, defined as the loss of skeletal muscle mass, may result in a reduced body mass within the range of the normal body mass index but can also occur in overweight individuals. A body mass index >30 kg·m^–2^ was excluded due to the high prevalence of comorbidities in this population.

**S2.2 Recruitment and Eligibility Assessment**

The participants were recruited via unaddressed letters distributed to randomly selected districts in the cantons of *Basel-Stadt* and *Basel-Landschaft*, Switzerland. The letters provided a detailed description of the study and its eligibility criteria, as well as a link to an online questionnaire created using the Research Electronic Data Capture platform. This questionnaire assessed the aforementioned criteria, as well as pre-exercise risk stratification [3]. Eligible individuals were subsequently interviewed by an exercise physiologist to verify their medical history. Final eligibility for study participation was confirmed on the day of the initial laboratory assessment. The physical examination performed by a physician included cardiac and pulmonary auscultation, assessment of joint function, evaluation of neurological symptoms, pain assessment, and evaluation of exercise tolerance. A substantial number of participants were excluded at this stage due to previously undiagnosed conditions or impairments identified during the examination.

**S2.3 Overview of Assessed Measures**

| **Table S1.** Overview of outcome variables, assessment methods, and assessment time points. | | | | | | | | | | |
| --- | --- | --- | --- | --- | --- | --- | --- | --- | --- | --- |
|  |  | **Measures and methods** | | |  | **Assessment time points** | | | | |
|  |  | *Outcome/Variable* | *Method/Instrument* | *Unit/Scale* |  | *Screening* | *Pre* | *W3* | *W12* | *Post* |
| **Screening** |  | Preliminary eligibility | Online questionnaire (including PAR-Q) | categorical/binary |  | ✓ |  |  |  |  |
|  |  | Medical history | Structured interview | categorical |  | ✓ |  |  |  |  |
|  |  | Clinical eligibility | Physical examination | categorical |  | ✓ |  |  |  |  |
| **Anthropometry** |  | Height | Stadiometer | cm |  |  | ✓ |  |  |  |
|  |  | Body mass | BIA | kg |  |  | ✓ |  |  |  |
| **Clinical** |  | Resting blood pressure | Manual sphygmomanometer | mmHg |  |  | ✓ |  |  | ✓ |
|  |  | Resting ECG | 12-lead ECG | – |  |  | ✓ |  |  | ✓ |
| **Body composition** |  | ALMI/ALMI | DXA | kg/kg·m^–2^ |  |  | ✓ |  |  | ✓ |
|  |  | Fat mass | DXA | kg |  |  | ✓ |  |  | ✓ |
|  |  | Body fat content | DXA | % |  |  | ✓ |  |  | ✓ |
| **Strength^*^** |  | IMTP strength | Force plate | N·kg⁻¹ |  |  | ✓ |  |  | ✓ |
|  |  | HGS | Dynamometer | kg·m^–2^ |  |  | ✓ |  |  | ✓ |
| **Cardiorespiratory fitness** |  | V̇O_₂peak_ | CPET | mL·kg⁻¹·min⁻¹ |  |  | ✓ |  |  | ✓ |
|  |  | Peak power output | CPET | W |  |  | ✓ |  |  | ✓ |
|  |  | Peak heart rate | CPET + ECG | bpm |  |  | ✓ |  |  | ✓ |
|  |  | Time to exhaustion | CPET | min |  |  | ✓ |  |  | ✓ |
| **Lipid profile** |  | Total cholesterol | Fasting blood sample | mmol·L^-1^ |  |  | ✓ |  |  | ✓ |
|  |  | HDL cholesterol | Fasting blood sample | mmol·L^-1^ |  |  | ✓ |  |  | ✓ |
|  |  | LDL cholesterol | Fasting blood sample | mmol·L^-1^ |  |  | ✓ |  |  | ✓ |

| **Table S1.** (continued) | | | | | | | | | | |
| --- | --- | --- | --- | --- | --- | --- | --- | --- | --- | --- |
|  |  | Triglycerides | Fasting blood sample | mmol·L^-1^ |  |  | ✓ |  |  | ✓ |
| **Glucose metabolism** |  | Fasting glucose | Fasting blood sample | mmol·L^-1^ |  |  | ✓ |  |  | ✓ |
|  |  | HbA1c | Blood sample | % |  |  | ✓ |  |  | ✓ |
|  |  | OGTT glucose | OGTT | mmol·L^-1^ |  |  | ✓ |  |  |  |
|  |  | OGTT insulin | OGTT | µU·mL^-1^ |  |  | ✓ |  |  |  |
|  |  | OGTT C-peptide | OGTT | pmol·L^-1^ |  |  | ✓ |  |  |  |
| **Skeletal muscle tissue^°^** |  | Vastus lateralis muscle biopsy | Core needle biopsy | – |  |  | ✓ |  |  |  |
| **Psychological and self-reported outcomes** |  | Chronotype | MCTQ *(14 items)* | score |  |  | ✓ |  |  | ✓ |
|  |  | Socioeconomic status | *(6 items)* | score |  |  | ✓ |  |  | ✓ |
|  |  | Quality of life | SF-8 *(8 items)* | score |  |  | ✓ |  |  | ✓ |
|  |  | Perceived stress | PSS *(4 items)* | score |  |  | ✓ |  |  | ✓ |
|  |  | Sleep quality | PSQI *(17 items)* | score |  |  | ✓ |  |  | ✓ |
|  |  | Insomnia severity | ISI *(7 items)* | score |  |  | ✓ |  |  | ✓ |
|  |  | Exercise enjoyment | PACES | score |  |  |  | ✓^†^ | ✓^†^ |  |
|  |  | Appetite | VAS | score |  |  |  | ✓^†^ | ✓^†^ |  |
| **Behavioral monitoring** |  | Physical activity | Wrist accelerometer | derived |  |  | ✓ | ✓ | ✓ |  |
|  |  | Sleep quality and duration | Wrist accelerometer | derived |  |  | ✓ | ✓ | ✓ |  |
| **Dietary assessment** |  | Eating behavior | Remote food photography | derived |  |  | ✓ | ✓ | ✓ |  |
| *Abbreviations:* ALM/ALMI, appendicular lean mass/appendicular lean mass index; ANCOVA, analysis of covariance; BIA, bioelectrical impedance analysis; bpm, beats per minute; CPET, cardiopulmonary exercise testing; DXA, dual-energy X-ray absorptiometry; ECG, electrocardiogram; HbA1c, hemoglobin A1c; HDL, high-density lipoprotein; HGS, handgrip strength; IMTP, isometric mid-thigh pull; ISI, Insomnia Severity Index; LDL, low-density lipoprotein; MCTQ, Munich Chronotype Questionnaire; OGTT, oral glucose tolerance test; PACES, Physical Activity Enjoyment Scale; PAR-Q, Physical Activity Readiness Questionnaire; Post, post-intervention; Pre, pre-intervention; PSS, Perceived Stress Scale; PSQI, Pittsburgh Sleep Quality Index; QoL, quality of life; SF-8, 8-item Short Form Health Survey; VAS, visual analogue scale for retrospective appetite assessment; V̇O_₂peak_, peak oxygen uptake; W3, week three of training intervention; W12, week 12 of training intervention. ^*^Strength outcomes were assessed at pre- and post-intervention at four standardized times of day (08:00, 12:00, 16:00, and 20:00) before and after the intervention. **^°^**Vastus lateralis muscle biopsies were collected from a subset of participants who had voluntarily consented to the procedure. | | | | | | | | | | |

**S2.4 Detailed Description of Assessed Measures**

*Skeletal muscle strength*

Strength performance assessments included the IMTP and HGS test. At least 24 hours before the first strength test, participants performed a familiarization trial to minimize potential learning effects. This involved performing two maximal repetitions of both the IMTP and HGS test. Before each testing session, participants performed a standardized 5-minute warm-up on a rowing ergometer. To ensure further standardization, the force plate was calibrated before each trial and, prior to testing, participants were verbally encouraged to exert maximal strength during testing.

Whole-body strength was assessed using IMTP testing [4,5]. A force plate (Leonardo Mechanography GF®, Novotec Medical GmbH, Pforzheim, Germany) was placed beneath a custom-made rack. The bar height was individually adjusted to position participants at a knee and hip angle of approximately 110°, while ensuring they maintained extended arms and a straight back. Joint angles were measured using a hand-held goniometer. Participants' feet were placed hip-width apart and centered under the bar, with their thighs lightly touching the bar. The shoulders were positioned above or slightly behind the bar, and a fixed gaze on the wall was maintained throughout testing. The bar height was recorded during the familiarization trial to ensure identical positioning during subsequent pre- and post-intervention assessments. Participants were instructed to push their feet into the ground as hard as possible while simultaneously pulling the bar upwards with maximal effort, increasing it gradually rather than explosively. They were asked to maintain maximal effort until prompted to stop (after approximately five seconds). Two consecutive trials were performed, and the higher value was used for evaluation. From the strength-time curve, relative maximal isometric strength normalized to body mass (N·kg⁻¹) was derived as the primary outcome.

HGS was measured using a handheld dynamometer (Leonardo Mechanography GF®, Novotec Medical GmbH, Pforzheim, Germany) [6,7]. Participants stood upright holding the device with the shoulders, forearms, and wrists maintained in a neutral (0°) position and the elbows fully extended (0°). Throughout the testing, a fixed gaze was maintained on a marked spot on the wall at a consistent height. Participants were instructed to exert maximal effort as rapidly as possible and maintain it until prompted to stop (after approximately five seconds). Two consecutive trials were performed on the dominant side, and the higher value was used for analysis. From the recorded strength-time curve, relative maximal HGS (kg·m⁻²) was derived as the primary outcome by normalizing grip strength to height squared to account for interindividual differences in body size.

To capture overall daily strength rather than performance at a single time point, mean daily scores for relative IMTP strength and relative HGS were calculated for the pre- and post-intervention assessments. For each participant, the maximum value obtained at each of the four measurement times (08:00, 12:00, 16:00, and 20:00) was identified and averaged to derive the daily mean. This procedure was applied identically to the IMTP and HGS outcomes.

*Body composition*

Body composition was assessed by dual-energy X-ray absorptiometry using a GE Lunar iDXA® machine (GE Lunar Inc., Madison, WI; software version 13.10). Absolute and relative body compositions were obtained in the morning following an overnight fast. Participants remained in the supine position throughout the assessment. ALMI (kg·m⁻²) was calculated as the sum of lean soft tissue mass of the upper and lower extremities normalized to height squared, a validated surrogate marker of skeletal muscle mass that accounts for differences in body size [8,9]. Body fat content (%) was included solely as a descriptive participant characteristic.

*Cardiopulmonary Exercise Testing*

Participants underwent maximal cardiopulmonary exercise testing on an electromagnetically braked cycle ergometer (Ergoselect 200; Ergoline, Bitz, Germany) using three different ramp protocols. Each protocol consisted of a 5-minute warm-up at 10, 20, or 50 W, followed by a continuous ramp increase of 10, 15, or 20 W per minute, and concluded with a 5-minute recovery at the initial workload. The protocol was individually based on the participant’s predicted peak oxygen uptake (estimated from sex, age, body mass, and height) to achieve a test duration of 6–18 minutes.

Pulmonary gas exchange was measured continuously breath-by-breath (MetaMax 3B; Cortex Biophysik GmbH, Leipzig, Germany) and averaged over 10-second intervals for analysis. Peak oxygen uptake was defined as the highest consecutive 30-second value of oxygen uptake_,_ while the maximal respiratory exchange ratio was defined as the highest 10-second value obtained during the ramp phase. Heart rate was continuously recorded using a 12-channel electrocardiogram (Custo med GmbH, Ottobrunn, Germany) and averaged over 10-second intervals. Peak heart rate was defined as the highest 10-second value recorded during the ramp phase. Peak power output was defined as the highest workload achieved at maximal voluntary exhaustion, and time to exhaustion was defined as the elapsed time from the start of the ramp phase until maximal voluntary exhaustion.

Ratings of perceived exertion were assessed using the Borg 1-10 scale at rest, after the warm-up, every three minutes during the exercise testing, at maximal voluntary exhaustion, and after three minutes of recovery. To identify physiological indications for test termination, blood pressure was measured using a manual sphygmomanometer at rest, after the warm-up, every three minutes during the exercise, at maximal voluntary exhaustion, and after three minutes of recovery.

Participants were verbally encouraged before and throughout testing to exercise until maximal exhaustion. They were allowed to choose their preferred pedalling cadence, provided it exceeded 60 revolutions per minute. Prior to each test, volume and two-point gas concentration calibration were performed on the respective metabolic cart.

*Blood Pressure Assessment*

Resting blood pressure was assessed following a standardized protocol. After at least ten minutes of seated rest, three consecutive blood pressure measurements were obtained, each separated by 60 seconds. The mean of the three measurements was used for analysis, in accordance with current recommendations [10].

*Fasting blood samples*

Fasting blood samples were obtained by venipuncture from the cubital fossa of the right or left arm. Participants followed the standardized pre-testing preparation procedures described in the main Methods section; for these measures, an overnight fast was additionally required. Analyses included total cholesterol, high-density lipoprotein, cholesterol, low-density lipoprotein cholesterol, triglycerides, fasting glucose, and hemoglobin A1c.

*Oral Glucose Tolerance Test*

Following the collection of fasting blood samples, participants underwent a 2-hour oral glucose tolerance test. After ingestion of a 75 g dextrose solution dissolved in 300 mL of water, venous blood samples were drawn at 10-, 20-, 30-, 60-, 90- and 120-minutes. Serum glucose, insulin and C-peptide concentrations were analyzed at each time point.

*Skeletal Muscle Biopsies*

Skeletal muscle biopsies were optional and were performed on a small subgroup of participants only. Biopsy samples were obtained from the vastus lateralis muscle at rest using the Magnum MG1522 biopsy device with a 16-gauge disposable core needle (Bard Medica SA, Oberrieden, Switzerland), a technique that has been shown to be valid and well tolerated [11].

*Questionnaires*

Participants completed a set of validated questionnaires assessing physical readiness, circadian preference, psychosocial health, sleep, stress, and behavioral responses to the intervention. Prior to study participation, the Physical Activity Readiness Questionnaire [3] was administered for pre-exercise screening. At the pre- and post-intervention assessments, chronotype was assessed using the Munich Chronotype Questionnaire [12], health-related quality of life using the Short-Form-8 Health Survey [13], sleep quality and sleep disturbances using the Pittsburgh Sleep Quality Index [14] and the Insomnia Severity Index [15], and perceived stress using the Perceived Stress Scale [16]. During the 12-week intervention period, participants in the training groups additionally completed the Physical Activity Enjoyment Scale [17], and retrospective visual analogue scales to assess appetite. An overview of all questionnaires, including the number of items, scoring, and assessment time points, is provided in Table S1.

**S2.5 Detailed Description of Monitoring of Confounding Factors**

*Physical Activity and Sleeping Behavior*

Participants were instructed to wear a GENEActiv triaxial accelerometer (Activinsights Ltd., Kimbolton, UK) on their non-dominant wrist for 24 hours a day over eight consecutive days. Assessments were conducted at pre-intervention assessment and during weeks three and 12 of the intervention or control phase. Data were sampled at 87.5 Hz, with a minimum wear time of 14 hours per day required for validity [18]. For inclusion in the analysis, participants were required to provide at least four valid weekdays (Monday–Friday) and one valid weekend day (Saturday or Sunday) [19].

*Eating Behavior*

Eating behavior was assessed using the remote food photography method [20]. Participants were instructed to record all meals and snacks consumed over four consecutive days before the intervention and at week three and week 12 of the intervention or control phase. Prior to data collection, participants received standardized instructions on photographing eating occasions using a smartphone. A reference card of known dimensions was provided to facilitate portion size estimation. Participants were instructed to send each image directly to the study team, along with written information on meal composition and the time of consumption. To maximize compliance, reminder messages were issued, and participants who failed to submit records for at least three meals per day were contacted by telephone for verification.

**S2.6 Statistical Considerations**

*Analysis Sample*

Analyses were conducted on the final sample of participants with available outcome data, as illustrated in Figure 1. No missing data were present.

*Statistical Analysis of Time-of-Day Training Effects*

Time-of-day was not independently randomized but occurred within the peak and trough randomization strata. To examine time-of-day effects, randomization group and training time of day were combined into a single categorical variable with nine levels: peak_morning, peak_noon, peak_afternoon, peak_evening, trough_morning, trough_noon, trough_afternoon, trough_evening, and control. Estimated marginal means were calculated for each randomization–time-of-day combination. Overall time-of-day comparisons (e.g., morning versus noon) were derived using pre-specified model-based contrasts that averaged allocation-specific differences (e.g., peak_morning versus peak_noon; trough_morning versus trough_noon). This process estimated time-of-day effects independent of the original peak–trough randomization. Comparisons involving the control group were constructed accordingly.

*Restricted Cubic Spline Specification*

Continuous covariates were modeled using restricted cubic splines with four knots to allow for potential nonlinear associations. Knot locations were placed at suitable quantiles of the respective variable distributions. Age (61.0, 64.0, 68.8, 78.0 years) and body mass (53.30, 66.32, 78.82, 93.60 kg) were modeled in their original units in both analyses. In the analysis of effect sizes, pre-intervention IMTP strength (18.70, 22.76, 25.76, 31.23 N·kg⁻¹), HGS (9.21, 11.27, 13.42, 17.02 kg·m⁻^2^), ALMI (5.66, 6.86, 8.04, 9.07 kg·m⁻^2^), and workload (63982.20, 95789.81, 121270.13, 163729.70 Σ kg·repetitions) were modeled in their original units. In the analysis of standardized effect sizes, these variables were z-standardized prior to modeling, with corresponding knot locations for IMTP strength (–1.58, –0.49, 0.32, 1.80), HGS (–1.39, –0.53, 0.36, 1.87), ALMI (–1.55, –0.49, 0.54, 1.44), and workload (–1.35, –0.42, 0.33, 1.57).

*R Packages*

Packages used for the statistical analyses were emmeans (version 2.0.0) [21], rms (version 8.1-0) [22], ggplot2 (version 4.0.1) [23], and dplyr (version 1.1.4) [24].

**S3 Results**

**S3.1 Baseline Characteristics**

| **Table S2.** Baseline characteristics of the time-of-day-specific groups. | | | | |
| --- | --- | --- | --- | --- |
| **Characteristics** | **Morning** | **Noon** | **Afternoon** | **Evening** |
| Sex, *male/female (n)* | 10/7 | 15/12 | 10/7 | 15/11 |
| Age *(years)* | 70 (5)  [62–78] | 66 (5)  [60–78] | 67 (5)  [60–78] | 66 (3)  [60–73] |
| Body mass *(kg)* | 70.4 (11.9)  [52.6–87.9] | 73.7 (12.8)  [54.5–99.4] | 76.7 (15.3)  [52.4–97.1] | 71.3 (12.0)  [50.2–97.6] |
| Body height *(cm)* | 171 (8)  [155–182] | 172 (8)  [159–190] | 171 (10)  [156–189] | 172 (9)  [156–186] |
| BMI *(kg*·*m^–2^)* | 23.8 (2.7)  [19.6–28.3] | 24.7 (2.9)  [21.1–29.8] | 25.8 (2.8)  [19.4–28.9] | 23.9 (2.7)  [19.0–28.7] |
| Body fat content *(%)* | 29.6 (7.6)  [18.8–43.7] | 32.6 (5.9)  [18.6–42.7] | 33.8 (5.0)  [26.3–40.6] | 29.7 (7.8)  [16.5–46.7] |
| Data are mean (standard deviation) [range] unless noted otherwise.  *Abbreviations:* Afternoon, intervention group training at 16:00; BMI, body mass index; Evening, intervention group training at 20:00; Morning, intervention group training at 08:00; Noon, intervention group training at 12:00. | | | | |

**S3.2 Reasons for Withdrawal by Time-of-Day-Specific Groups**

The following section provides a detailed breakdown of the specific circumstances surrounding study withdrawals across the time-of-day-specific training groups. Dropouts occurred both before intervention onset and during post-intervention assessments, with heterogenous reasons across groups (Figure 1). No withdrawals occurred in the morning group. In the noon group, two females and one male withdrew, all from the peak randomization group, due to injuries unrelated to the intervention (n=2) or musculoskeletal complaints during post-intervention assessments (n=1). In the afternoon group, two females and three males withdrew, due to time commitment issues (n=1), medical conditions unrelated to the study (n=1), or musculoskeletal complaints (n=3). In the evening group, five females and one male withdrew, predominantly due to musculoskeletal complaints during post-intervention assessments (n=5) or medical conditions unrelated to the study (n=1).

**S3.3 Progression of Training Volume Across the Training Intervention**

**Fig S1** Exercise-specific volume (A–E) and total session volume (F) across resistance training sessions for the morning (training at 08:00), noon (training at 12:00), afternoon (training at 16:00), and evening (training at 20:00) groups. Exercise-specific volume was calculated as load × repetitions; for bilateral exercises (leg press and cross body cable row), volume was calculated for a single side only. Total session volume was computed as the sum of all exercise-specific volumes per session. Solid lines depict group means, shaded areas indicate variability, and thin lines represent individual participant trajectories.

**S3.4 Progression of Absolute Training Loads Across the Training Intervention**

**Fig S2** Absolute training loads for leg press (A), chest press (B), deadlift (C), cross body cable row (D), and back squat (E) across time points for the peak (training at their individual peak time of day) and trough (training at their individual trough time of day) groups. Time points include T1 (first resistance training session following the 2-week familiarization period), T2 (midpoint of the resistance training phase, corresponding to 50% of each participant’s individual maximum number of completed resistance training sessions), and T3 (final resistance training session). Individual participant trajectories are shown with dashed lines, with boxplots representing the distribution of training loads at each time point.

**Fig S3** Absolute training loads for leg press (A), chest press (B), deadlift (C), cross body cable row (D), and back squat (E) are shown across time points for the morning (training at 08:00), noon (training at 12:00), afternoon (training at 16:00), and evening (training at 20:00) groups. Time points include T1 (first resistance training session following the 2-week familiarization period), T2 (midpoint of the resistance training phase, corresponding to 50% of each participant’s individual maximum number of completed resistance training sessions), and T3 (final resistance training session). Individual participant trajectories are shown with dashed lines, with boxplots representing the distribution of training loads at each time point.

**S3.5 Diurnal Variation in Strength Performance**

**Fig S4** Diurnal variation in relative isometric mid-thigh pull (IMTP) strength (A) and relative handgrip strength (HGS) (B) across test times of day. Data are shown for all participants, for peak–trough–control randomization groups, and for time-of-day-specific groups. Boxplots depict the distribution of values at each test times of day, with individual participant trajectories shown as dashed lines. Test times of day are morning (M), noon, (N), afternoon (A), and evening (E).

**S3.6 Training-Induced Skeletal Muscle Adaptations**

**Fig S5** Relative mean daily isometric mid-thigh pull (IMTP) strength (A), relative mean daily handgrip strength (HGS) (B), and appendicular lean mass index (ALMI) (C) shown pre- and post-intervention, stratified by peak–trough randomization and sex. 'Peak' and 'Trough' denote intervention groups training at their individual peak or trough time of day, respectively. Time points are 'Pre' (before the training intervention or control phase) and 'Post' (after the training intervention or control phase). 'Strength_daily_' represents the mean of each participant’s maximal strength values obtained at four measurement times (08:00, 12:00, 16:00, and 20:00). Dashed lines depict individual participant changes between time points; boxplots summarize the distribution at each time point.

**S3.7 Between-Group Differences in Skeletal Muscle Adaptations**

| **Table S3**. Baseline-adjusted between-group differences reported as effect sizes derived from analyses of covariance. | | | | | | | | | | | | |
| --- | --- | --- | --- | --- | --- | --- | --- | --- | --- | --- | --- | --- |
|  |  | **IMTP strength_daily_** | | |  | **HGS_daily_** | | |  | **ALMI** | | |
|  |  | Model 1 | Model 2 | Model 3 |  | Model 1 | Model 2 | Model 3 |  | Model 1 | Model 2 | Model 3 |
| **Comparison** |  | ES  [95% CI] | ES  [95% CI] | ES  [95% CI] |  | ES  [95% CI] | ES  [95% CI] | ES  [95% CI] |  | ES  [95% CI] | ES  [95% CI] | ES  [95% CI] |
| Peak–trough–control randomization groups | | | | | | | | | | | | |
| *Peak – Trough* |  | 0.07  [–0.77–0.90] | 0.06  [–0.76–0.88] | –0.05  [–0.71–0.61] |  | –0.22  [–0.68–0.24] | –0.21  [–0.68–0.26] | –0.17  [–0.58–0.23] |  | 0.04  [–0.08–0.16] | 0.03  [–0.09–0.15] | 0.03  [–0.07–0.14] |
| *Peak – Control* |  | 0.52  [–0.51–1.55] | 0.65  [–0.39–1.69] | NA |  | –0.17  [–0.72–0.38] | –0.03  [–0.61–0.55] | NA |  | 0.09  [–0.06–0.24] | 0.07  [–0.08–0.23] | NA |
| *Trough – Control* |  | 0.45  [–0.54–1.44] | 0.59  [–0.42–1.60] | NA |  | 0.05  [–0.48–0.58] | 0.18  [–0.38–0.74] | NA |  | 0.05  [–0.09–0.20] | 0.04  [–0.10–0.19] | NA |
| Time-of-day-specific allocation groups | | | | | | | | | | | | |
| *Morning – Noon* |  | 1.08  [–1.81–3.97] | 1.39  [−1.63–4.41] | 1.27  [–1.73–4.26] |  | 0.39 [−1.19–1.96] | 0.50 [−1.16–2.16] | 0.45  [–1.22–2.13] |  | –0.16  [–0.56–0.25] | –0.13  [–0.56–0.29] | –0.13  [–0.56–0.31] |
| *Morning – Afternoon* |  | 2.40  [–0.81–5.61] | 2.40  [−0.84–5.64] | 2.45  [–0.76–5.66] |  | −0.43  [−2.19–1.32] | −0.85  [−2.71–1.01] | –0.86  [–2.74–1.02] |  | –0.19  [–0.66–0.29] | –0.15  [–0.63–0.33] | –0.14  [–0.63–0.34] |
| *Morning – Evening* |  | 0.96  [–1.98–3.90] | 0.94  [−2.04–3.93] | 1.32  [–1.76–4.41] |  | 0.64 [−0.94–2.22] | 0.50  [−1.14–2.15] | 0.47  [–1.27–2.20] |  | –0.04  [–0.45–0.37] | –0.03  [–0.45–0.39] | 0.01  [–0.44–0.46] |
| *Noon – Afternoon* |  | 1.32  [–1.82–4.46] | 1.02  [−2.10–4.14] | 1.18  [–1.92–4.29] |  | −0.82  [−2.56–0.91] | −1.35  [−3.14–0.43] | –1.31  [–3.13–0.50] |  | –0.03  [–0.50–0.43] | –0.02  [–0.49–0.45] | –0.02  [–0.50–0.46] |
| *Noon – Evening* |  | –0.12  [–3.03–2.78] | −0.45  [−3.43–2.53] | 0.06  [–3.03–3.14] |  | 0.25 [−1.30–1.81] | 0.00  [−1.61–1.62] | 0.01  [–1.71–1.74] |  | 0.11  [–0.28–0.51] | 0.10  [–0.32–0.52] | 0.14  [–0.31–0.59] |
| *Afternoon – Evening* |  | –1.45  [–4.62–1.73] | −1.46  [−4.62–1.70] | –1.12  [–4.35–2.10] |  | 1.08  [−0.64–2.79] | 1.35 [−0.43–3.14] | 1.33  [–0.52–3.17] |  | 0.15  [–0.32–0.62] | 0.12  [–0.35–0.59] | 0.16  [–0.34–0.65] |
| *Morning – Control* |  | 1.05  [–0.39–2.49] | 1.19  [−0.26–2.64] | NA |  | 0.05  [−0.71–0.81] | 0.16  [−0.62–0.94] | NA |  | –0.01  [–0.20–0.19] | –0.01  [–0.21–0.19] | NA |
| *Noon – Control* |  | 0.51  [–0.90–1.92] | 0.50  [−0.93–1.92] | NA |  | −0.14  [−0.88–0.60] | −0.09  [−0.86–0.68] | NA |  | 0.07  [–0.12–0.26] | 0.05  [–0.15–0.25] | NA |
| *Afternoon – Control* |  | –0.15  [–1.71–1.41] | –0.01  [−1.58–1.56] | NA |  | 0.27 [−0.56–1.10] | 0.59 [−0.31–1.48] | NA |  | 0.09  [–0.14–0.31] | 0.06  [–0.17–0.29] | NA |
| *Evening – Control* |  | 0.57  [–0.81–1.95] | 0.72  [−0.72–2.17] | NA |  | −0.27  [−1.02–0.47] | −0.09  [−0.89–0.71] | NA |  | 0.01  [–0.18–0.21] | 0.00  [–0.20–0.20] | NA |
| *Abbreviations:* Afternoon, intervention group training at 16:00; ALMI, appendicular lean mass index; Control, control group maintaining their habitual lifestyle; ES, effect size (adjusted between-group difference, expressed in original units [N·kg⁻¹ for IMTP strength, kg·m⁻^2^ for HGS and ALMI]; Evening, intervention group training at 20:00; HGS, handgrip strength; IMTP, isometric mid-thigh pull; Model 1, including baseline values and group allocation; Model 2, additionally included age and sex for the outcomes IMPT strength and ALMI, and age, sex, and body mass for the outcome HGS; Model 3, additionally including training volume; Morning, intervention group training at 08:00; NA, not applicable; Noon, intervention group training at 12:00; Peak, intervention group training at their individual peak time of day; Strength_daily_, mean of each participant’s maximal strength values obtained at four measurement times (08:00, 12:00, 16:00, and 20:00); Trough, intervention group training at their individual trough time of day; 95% CI, 95% confidence interval. | | | | | | | | | | | | |

| **Table S4**. Baseline-adjusted between-group differences reported as standardized effect sizes derived from analyses of covariance. | | | | | | | | | | | | |
| --- | --- | --- | --- | --- | --- | --- | --- | --- | --- | --- | --- | --- |
|  |  | **IMTP strength_daily_** | | |  | **HGS_daily_** | | |  | **ALMI** | | |
|  |  | Model 1 | Model 2 | Model 3 |  | Model 1 | Model 2 | Model 3 |  | Model 1 | Model 2 | Model 3 |
| **Comparison** |  | ES  [95% CI] | ES  [95% CI] | ES  [95% CI] |  | ES  [95% CI] | ES  [95% CI] | ES  [95% CI] |  | ES  [95% CI] | ES  [95% CI] | ES  [95% CI] |
| Peak–trough–control randomization groups | | | | | | | | | | | | |
| *Peak – Trough* |  | 0.02  [–0.21–0.24] | 0.02  [–0.21–0.24] | –0.01  [–0.19–0.17] |  | –0.09  [–0.28–0.10] | –0.09  [–0.28–0.11] | –0.07  [–0.24–0.10] |  | 0.03  [–0.08–0.14] | 0.03  [–0.08–0.14] | 0.03  [–0.07–0.13] |
| *Peak – Control* |  | 0.14  [–0.14–0.42] | 0.18  [–0.11–0.46] | NA |  | –0.07  [–0.30–0.16] | –0.01  [–0.25–0.23] | NA |  | 0.08  [–0.05–0.22] | 0.07  [–0.07–0.20] | NA |
| *Trough – Control* |  | 0.12  [–0.15–0.39] | 0.16  [–0.11–0.44] | NA |  | 0.02  [–0.20–0.24] | 0.08  [–0.16–0.31] | NA |  | 0.05  [–0.08–0.18] | 0.04  [–0.09–0.17] | NA |
| Time-of-day-specific allocation groups | | | | | | | | | | | | |
| *Morning – Noon* |  | 0.30  [–0.49–1.08] | 0.38  [−0.44–1.20] | 0.37  [–0.40–1.15] |  | 0.16  [−0.49–0.82] | 0.21  [−0.48–0.90] | 0.07  [–0.63–0.78] |  | –0.14  [–0.50–0.22] | –0.12  [–0.50–0.26] | –0.19  [–0.57–0.20] |
| *Morning – Afternoon* |  | 0.66  [–0.22–1.53] | 0.66  [−0.23–1.54] | 0.66  [–0.16–1.47] |  | −0.18  [−0.91–0.55] | −0.35  [−1.12–0.42] | –0.35  [–1.12–0.41] |  | –0.17  [–0.60–0.26] | –0.13  [–0.56–0.29] | –0.14  [–0.56–0.28] |
| *Morning – Evening* |  | 0.26  [–0.54–1.06] | 0.26  [−0.56–1.07] | 0.31  [–0.48–1.10] |  | 0.27  [−0.39–0.92] | 0.21  [−0.47–0.89] | 0.15  [–0.56–0.86] |  | –0.04  [–0.40–0.33] | –0.03  [–0.40–0.35] | –0.01  [–0.40–0.38] |
| *Noon – Afternoon* |  | 0.36  [–0.49–1.22] | 0.28  [−0.57–1.13] | 0.28  [–0.53–1.10] |  | −0.34  [−1.06–0.38] | −0.56  [−1.30–0.18] | –0.43  [–1.19–0.33] |  | –0.03  [–0.45–0.39] | –0.01  [–0.43–0.41] | 0.05  [–0.37–0.47] |
| *Noon – Evening* |  | –0.03  [–0.83–0.76] | −0.12  [−0.93–0.69] | –0.07  [–0.86–0.73] |  | 0.11  [−0.54–0.75] | 0.00  [−0.67–0.67] | 0.08  [–0.64–0.79] |  | 0.10  [–0.25–0.45] | 0.09  [–0.28–0.47] | 0.18  [–0.22–0.58] |
| *Afternoon – Evening* |  | –0.39  [–1.26–0.47] | −0.40  [−1.26–0.46] | –0.35  [–1.17–0.47] |  | 0.45  [−0.27–1.16] | 0.56  [−0.18–1.31] | 0.50  [–0.25–1.26] |  | 0.13  [–0.28–0.55] | 0.11  [–0.31–0.53] | 0.13  [–0.30–0.56] |
| *Morning – Control* |  | 0.29  [–0.11–0.68] | 0.33  [−0.07–0.72] | NA |  | 0.02  [−0.30–0.34] | 0.07  [−0.26–0.39] | NA |  | –0.01  [–0.18–0.17] | –0.01  [–0.19–0.17] | NA |
| *Noon – Control* |  | 0.14  [–0.25–0.52] | 0.14  [−0.25–0.52] | NA |  | −0.06  [−0.37–0.25] | −0.04  [−0.36–0.28] | NA |  | 0.06  [–0.11–0.23] | 0.05  [–0.13–0.22] | NA |
| *Afternoon – Control* |  | –0.04  [–0.47–0.38] | 0.00  [−0.43–0.42] | NA |  | 0.11  [−0.23–0.46] | 0.24  [−0.13–0.62] | NA |  | 0.08  [–0.12–0.28] | 0.05  [–0.15–0.26] | NA |
| *Evening – Control* |  | 0.16  [–0.22–0.53] | 0.20  [−0.20–0.59] | NA |  | −0.11  [−0.42–0.20] | −0.04  [−0.37–0.29] | NA |  | 0.01  [–0.16–0.19] | 0.00  [–0.18–0.18] | NA |
| *Abbreviations:* Afternoon, intervention group training at 16:00; ALMI, appendicular lean mass index; Control, control group maintaining their habitual lifestyle; ES, standardized effect size (expressed relative to the baseline standard deviation of the respective outcome [unitless]; Evening, intervention group training at 20:00; HGS, handgrip strength; IMTP, isometric mid-thigh pull; Model 1, including baseline values and group allocation; Model 2, additionally included age and sex for the outcomes IMPT strength and ALMI, and age, sex, and body mass for the outcome HGS; Model 3, additionally including training volume; Morning, intervention group training at 08:00; NA, not applicable; Noon, intervention group training at 12:00; Peak, intervention group training at their individual peak time of day; Strength_daily_, mean of each participant’s maximal strength values obtained at four measurement times (08:00, 12:00, 16:00, and 20:00); Trough, intervention group training at their individual trough time of day; 95% CI, 95% confidence interval. | | | | | | | | | | | | |

**S3.8 Time-of-Day Specificity of Strength**

**Fig S6** Pre–post changes in relative isometric mid-thigh pull (IMTP) strength (A) and relative handgrip strength (HGS) (B) stratified by time-of-day-specific allocation and test times of day. The figure illustrates whether participants who trained at a given time of day (Morning, Noon, Afternoon, or Evening) showed larger improvements when tested at the corresponding time of day. The control group (Control), which maintained their habitual lifestyle, was also included in the analysis. Boxplots representing the distribution of values at each time point. Test times of day are morning (M), noon, (N), afternoon (A), and evening (E).

**References**

1. Walton RG, Dungan CM, Long DE, et al. Metformin blunts muscle hypertrophy in response to progressive resistance exercise training in older adults: A randomized, double-blind, placebo-controlled, multicenter trial: The MASTERS trial. *Aging Cell.* 2019;18:e13039. doi:10.1111/acel.13039.
2. Kulkarni AS, Peck BD, Walton RG, et al. Metformin alters skeletal muscle transcriptome adaptations to resistance training in older adults. *Aging.* 2020;12:19852–66. doi:10.18632/aging.104096.
3. Shephard RJ. PAR-Q, Canadian Home Fitness Test and exercise screening alternatives. *Sports Med.* 1988;**5**:185–95. doi:10.2165/00007256-198805030-00005
4. Grgic J, Scapec B, Mikulic P, Pedisic Z. Test-retest reliability of isometric mid-thigh pull maximum strength assessment: a systematic review. *Biol Sport*. 2022;**39**:407–14. doi:10.5114/biolsport.2022.106149
5. Comfort P, Dos’Santos T, Beckham GK, Stone MH, Guppy SN, Haff GG. Standardization and methodological considerations for the isometric midthigh pull. *Strength Cond J.* 2019;**41**:57–79. doi:10.1519/SSC.0000000000000433
6. Wang C-Y, Chen L-Y. Grip strength in older adults: test-retest reliability and cutoff for subjective weakness of using the hands in heavy tasks. *Arch Phys Med Rehabil*. 2010;**91**:1747–51. doi:10.1016/j.apmr.2010.07.225
7. Balogun JA, Akomolafe CT, Amusa LO. Grip strength: effects of testing posture and elbow position. *Arch Phys Med Rehabil.* 1991;**72**:280–3.
8. Kim J, Wang Z, Heymsfield SB, Baumgartner RN, Gallagher D. Total-body skeletal muscle mass: estimation by a new dual-energy X-ray absorptiometry method*. Am J Clin Nutr*. 2002;**76**:378–83. doi:10.1093/ajcn/76.2.378
9. Heymsfield SB, Adamek M, Gonzalez MC, Jia G, Thomas DM. Assessing skeletal muscle mass: historical overview and state of the art. *J Cachexia Sarcopenia Muscle*. 2014;**5**:9–18. doi:10.1007/s13539-014-0130-5
10. Whelton PK, Carey RM, Mancia G, Kreutz R, Bundy JD, Williams B. Harmonization of the American College of Cardiology/American Heart Association and European Society of Cardiology/European Society of Hypertension blood pressure/hypertension guidelines: comparisons, reflections, and recommendations. *Circulation.* 2022;**146**:868–77. doi:10.1161/CIRCULATIONAHA.121.054602
11. Newmire DE, Willoughby DS. The skeletal muscle microbiopsy method in exercise and sports science research: a narrative and methodological review. *Scand J Med Sci Sports.* 2022;**32**:1550–68. doi:10.1111/sms.14215
12. Roenneberg T, Kuehnle T, Juda M, et al. Epidemiology of the human circadian clock. *Sleep Med Rev.* 2007;**11**:429–38. doi:10.1016/j.smrv.2007.07.005
13. Ellert U, Lampert T, Ravens-Sieberer U. [Measuring health-related quality of life with the SF-8. Normal sample of the German population]. *Bundesgesundheitsblatt Gesundheitsforschung Gesundheitsschutz.* 2005;**48**:1330–7. doi:10.1007/s00103-005-1168-5
14. Buysse DJ, Reynolds CF 3rd, Monk TH, Berman SR, Kupfer DJ. The Pittsburgh Sleep Quality Index: a new instrument for psychiatric practice and research. *Psychiatry Res.* 1989;**28**:193–213. doi:10.1016/0165-1781(89)90047-4
15. Morin CM, Belleville G, Bélanger L, Ivers H. The Insomnia Severity Index: psychometric indicators to detect insomnia cases and evaluate treatment response. *Sleep.* 2011;**34**:601–8. doi:10.1093/sleep/34.5.601
16. Cohen S, Kamarck T, Mermelstein R. A global measure of perceived stress. *J Health Soc Behav.* 1983;**24**:385–96. doi:10.2307/2136404
17. Jekauc D, Nigg C, Nigg CR, et al. Measurement properties of the German version of the Physical Activity Enjoyment Scale for adults. *PloS One*. 2020;15:e0242069. doi:10.1371/journal.pone.0242069
18. Herrmann SD, Barreira TV, Kang M, Ainsworth BE. Impact of accelerometer wear time on physical activity data: a NHANES semisimulation data approach. *Br J Sports Med.* 2014;**48**:278–82. doi:10.1136/bjsports-2012-091410
19. Schwendinger F, Wagner J, Infanger D, Schmidt-Trucksäss A, Knaier R. Methodological aspects for accelerometer-based assessment of physical activity in heart failure and health. *BMC Med Res Methodol.* 2021;**21**:251. doi:10.1186/s12874-021-01350-6
20. Martin CK, Han H, Coulon SM, Allen HR, Champagne CM, Anton SD. A novel method to remotely measure food intake of free-living individuals in real time: the remote food photography method. *Br J Nutr.* 2009;**101**:446–56. doi:10.1017/S0007114508027438
21. Lenth R, Piaskowski J. emmeans: estimated marginal means, aka least-squares means. R package version. 2025. doi:10.32614/CRAN.package.emmeans
22. Harrell Jr FE. rms: regression modeling strategies. R package version. 2025. doi:10.32614/CRAN.package.rms
23. Wickham H. ggplot2: elegant graphics for data analysis. New York, NY: Springer; 2016.
24. Wickham H, François R, Henry L, Müller K, Vaughan D. dplyr: a grammar of data manipulation. R package version. 2023. doi:10.32614/CRAN.package.dplyr
